# Race, Family Conflict and Suicidal Thoughts and Behaviors among 9–10-Year-Old American Children

**DOI:** 10.3390/ijerph18105399

**Published:** 2021-05-18

**Authors:** Shervin Assari, Shanika Boyce, Mohsen Bazargan, Cleopatra H. Caldwell

**Affiliations:** 1 Department of Urban Public Health, Charles R Drew University of Medicine and Science, Los Angeles, CA 90059, USA; Mohsenbazargan@cdrewu.edu; 2 Department of Family Medicine, Charles R Drew University of Medicine and Science, Los Angeles, CA 90059, USA; 3 Department of Pediatrics, Charles R Drew University of Medicine and Science, Los Angeles, CA 90059, USA; shanikaboyce@cdrewu.edu; 4 Department of Family Medicine, University of California, Los Angeles (UCLA), Los Angeles, CA 90095, USA; 5 Department of Health Behavior and Health Education, School of Public Health, University of Michigan, Ann Arbor, MI 48109-2029, USA; cleoc@umich.edu; 6 Center for Research on Ethnicity, Culture, and Health, School of Public Health, University of Michigan, Ann Arbor, MI 48109-2029, USA

**Keywords:** family relations, race, suicidal thoughts and behaviors, suicide, children

## Abstract

Family conflict is known to operate as a major risk factor for children’s suicidal thoughts and behaviors (STBs). However, it is unknown whether this effect is similar or different in Black and White children. Objectives: We compared Black and White children for the association between family conflict and STBs in a national sample of 9–10-year-old American children. Methods: This cross-sectional study used data from the Adolescent Brain Cognitive Development (ABCD) study. This study included 9918 White or Black children between the ages of 9 and 10 living in married households. The predictor variable was family conflict. Race was the moderator. The outcome variable was STBs, treated as a count variable, reflecting positive STB items that were endorsed. Covariates included ethnicity, sex, age, immigration status, family structure, parental education, and parental employment, and household income. Poisson regression was used for data analysis. Results: Of all participants, 7751 were Whites, and 2167 were Blacks. In the pooled sample and in the absence of interaction terms, high family conflict was associated with higher STBs. A statistically significant association was found between Black race and family conflict, suggesting that the association between family conflict and STBs is stronger in Black than White children. Conclusion: The association between family conflict and STBs is stronger in Black than White children. Black children with family conflict may be at a higher risk of STBs than White children with the same family conflict level. These findings align with the literature on the more significant salience of social relations as determinants of mental health of Black than White people. Reducing family conflict should be regarded a significant element of suicide prevention for Black children in the US.

## 1. Background

Social relations are considered salient determinants of mental health outcomes [1]. Conflicting relations are known to increase the risk of undesired mental health outcomes for populations and individuals [2]. One of the main outcomes that are shown to be under the influence of conflicting relations is suicidal thoughts and behaviors (STBs) in both adults [3] and children [4]. However, some growing research shows that social relationships may have differential associations with the mental health of White and Black people [5]. We are, however, not aware of any previous studies on differences between White and Black children regarding the associations between conflicting family relations and suicidal thoughts and behaviors (STBs) [6].

Multiple theoretical frameworks suggest a link between family conflict and mental health outcomes such as STBs in children. Social support theory by Cohen [7] and House [8] suggest that relationship quality is a salient determinant of health. Based on Lazarus’s stress theory [9], we expect conflicting relations to operate as a risk factor for various mental health outcomes across age groups [10]. However, Krause, Chatters, Assari, Lincoln, and others have shown that social relations may be more influential for Black than White individuals. In response to slavery, racism, and discrimination, Black communities may have turned to social relationships [11]. At the same time, due to multiple Jeopardy hypothesis, we expect the same risk factor to have a more significant detrimental effect on the mental health of Blacks than Whites, simply because Blacks are already exposed to a wide range of social stressors, so they have become more vulnerable to the very same risk factors [12]. Finally, based on minorities’ diminished returns (MDRs) theory [13,14,15], we would expect family-level risk and protective factors to show diminished associations with mental health outcomes for Black than White communities. This is in part because, for Blacks, structural racism, social stratification, and other higher-level adversities may reduce the degree by which a risk factor or a protective factor translates to outcome [13].

Close relations such as those in the family not only provide social support but also become a source of negative interactions, relations, and encounters, which have undesired effects [16,17]. Family conflict, defined as negative social interactions that occur between members of the family, has significant influences on children’s mental health [18,19]. Given that young children are highly dependent on their parents, and as the family is the primary place of child development [20], children can be vulnerable to the mental health effects of negative family relationships [20]. As shown by Bauermeister, bad tends to be stronger than good [21]. As a result, conflicting relations seem to have a larger undesired effect on children’s mental health than the protective effects of support and positive relations [22]. Extensive research has connected family conflict to undesired mental health outcomes such as depression, anxiety, substance use, and suicide [23]. However, very little is known about racial variation in the role of conflicting family relationships as a risk factor for children’s suicidal thoughts and behaviors (STBs).

There is limited research comparing White and Black children for the association between social relations and STBs. Traditionally, STBs are seen as a White, not a Black public health problem [24,25]. Historically, most research shows that in children, youth, and adults, Whites are more likely to commit suicide than Blacks [26,27,28]. Low STBs are attributed to the low acceptability of STBs in Black families [29,30] that is attributed to the high religiosity [29], supportive social relations [31,32,33], and resilience [34,35,36] in Black communities. This pattern is also described as the Black–White mental health paradox, and some have argued that Blacks may have flourished in the presence of adversities, while Whites have been ill-prepared to handle stress as a function of their social privilege [37,38,39]. More recently, however, an alarmingly increasing trend of STBs in Black children, particularly boys, has been observed [40]. The National Institutes of Health (NIH) has called Black youth suicide a national crisis.

While a wide range of other factors such as sex, age, SES, academic success, substance use, depression, and stress also matter [41], a recent study using the Adolescent Brain Cognitive Development (ABCD) study data showed that in 9–10-year-old American children, family conflict seems to be one of the most substantial STB risk factors [42]. The study, however, did not test racial and group differences in the relevance of family conflict as a correlate of STBs [42].

Research has shown that STBs may correlate differently to risk factors such as stress in White and Black children, youth, and adults [43,44]. In a study of 229 18–33-year-old young adults, racial groups reported what had precipitated their STBs [45]. Although, overall, suicidal ideation was most frequently precipitated by an intrapersonal factor, followed by an interpersonal factor, Whites reported less interpersonal precipitation before their STBs than Hispanic, Asian, and biracial young adults [45]. A growing literature shows that stress may have a differential association for Black and White individuals’ mental health. This is based on the observation that chronic stress is more common in the lives of Black than White individuals [46]. In this view, high levels of stress in the lives of Black families may have prepared them to better face additional stress or increased their vulnerability to additional stress [47]. Conflicting findings have been reported, providing support for both sides of the argument. For example, one study showed a stronger association between general stress and depression for Whites than Blacks [34], while another study showed a stronger association between financial stress and depression for Blacks than Whites [36].

More recently, some research has shown that relationship quality, including those negative, may be more consequential for the mental health of Black than White individuals. For instance, Lincoln and colleagues showed that Blacks are more vulnerable to mental health effects of positive and negative relationships than Whites [48]. Krause and colleagues have shown that social support in the church setting has more consequences for Blacks than Whites [49,50,51], and another study showed that secular and religious social support are both more strongly associated with depression in Black than White individuals [52]. The same finding is shown by other research as well [5,53]. However, this literature is mostly on adults or even older adults, and few studies were done among children. Given that tolerance and resilience toward stress develop over time [54], it is important not to assume that the same pattern would be seen for children and test the racial difference in the association between the relationship quality and mental health of White and Black children. However, even for children, all types of stress seem to be higher for Blacks than Whites, meaning that additional stress in the lives of Blacks is not absent in the lives of Black children [55]. So, it is likely that the Black–White difference in the correlation between various types of stress and mental health outcomes would be observable in children [56].

Understanding how racial groups differ in the correlation between family conflict, as a primary social risk factor, and STBs, may improve our capability for suicide prevention, diagnosis, and treatment in Black children [57]. Such results may inform policies, practices, and interventions that can target racial subgroups and focus on those most vulnerable to STBs. The information on racial differences in correlates of STBs may help tailor suicide prevention programs for diverse populations for Black children and their families [45]. Still, we know little about how race may interact with family conflict as an STB risk factor [58].

### Objectives

To address the paucity of research on the interactive effects of race and STB risk factors among American children, we tested racial differences in the association between family conflict and childhood STBs. To do so, we used a national sample of 9–10-year-old American children.

## 2. Methods

### 2.1. Design and Setting

This is a secondary analysis of the Adolescent Brain Cognitive Development (ABCD) study data [59,60]. The analysis only used wave 1 (baseline) data; thus, the study has a cross-sectional design.

### 2.2. Sample and Sampling

The ABCD study included children between the ages of 9 and 10. The ABCD children were enrolled from multiple cities across multiple states. Children were recruited into the ABCD study from 21 sites across 15 states. The primary strategy for sampling in the ABCD study was sampling through school systems [61]. In the current study, we only included White and Black males and females.

### 2.3. Outcome

The children′s STBs (at baseline) were calculated based on the participants′ responses to the items on various aspects of STBs (Table 1) [62,63]. STBs were treated as a count measure that potentially ranged from 0 to 22. This STB variable (sum score) had a Cronbach’s alpha of 0.756. This measure was measured using K-SADS. A fully structured interview was implemented to test the presence or absence of each of the items listed in Table 1.

### 2.4. Moderators

For race, Blacks were coded as 1, and Whites were coded as 0.

### 2.5. Predictor

The predictor variable was family conflict. Family conflict was measured using the nine items of the Family Environment Scale [64]. In this measure, children report negative aspects of their family relations. Items on the conflict subscale assess the extent of fighting, anger, criticism, competitiveness, yelling, and/or loss of temper within the family [65]. The measure provides a continuous score with a higher score indicating higher conflict in the family. Items of the Family Environment Scale are shown in Table 1. Cronbach’s alpha of the family conflict measure was beyond 0.681 overall and higher than 0.6 for both White and Black sub-samples. Some of the items required reverse coding, so the endorsement of all items would indicate conflicting relations. We calculated a mean score, so that missing response to any of the items would not interfere with our measurement.

### 2.6. Covariates

Covariates included immigration status, ethnicity, sex, age, family structure, parental education, parental employment, and household income. For sex, males were coded as 1, and females were coded as 0. Age was recorded in months. Parental education was a continuous measure ranging from 1 to 23, with a higher score indicating more schooling years. To calculate parental education, we considered the highest education attainment of both parents. Area-level median home value was driven based on zip codes by the ABCD study team.

### 2.7. Data Analysis

SPSS was used to analyze the data. Data were downloaded from the National Institutes of Health (NIH) National Data Archive (NDA) website. Mean (standard deviation; SD) and frequency (relative frequency; %) of all variables were described overall and for each race. We used independent sample *t*-test and chi-square tests to compare the study variables among races. For multivariable modeling, we ran Poisson regression models. As the participants were nested to families who were nested to sites, we calculated the intra-class correlation for our outcome. Our calculation showed a less than 0.05 intra-class correlation, which is minimal. As such, we did not apply mixed-effect or random-effect models. The predictor variables were family conflict and support. The moderator was race. The outcome variable was STBs, treated as a count variable, reflecting positive suicidal items endorsed. Covariates included sex, age, ethnicity, immigration status, parental education, parental employment, and household income. Multiple Poisson regression models were performed in the pooled sample in the absence and presence of family support/conflict by race interaction. Before we performed our models, we ruled out multi-collinearity between the study variables. We also explored the distribution of our predictors (a), outcomes (b), residuals (c), and quantiles (d). Beta coefficient (b), 95% confidence intervals (CI), standard error (SE), and *p*-value were reported for our model. A *p*-value equal to or less than 0.05 was significant.

### 2.8. Ethics

The ABCD study protocol received an Institutional Review Board (IRB) approval from several institutions, including but not limited to the University of California, San Diego (UCSD). All participating children provided assent. All participating parents signed an informed consent [66]. Our study was exempt from a full IRB review.

## 3. Results

### 3.1. Descriptive Data Overall

This study included 9918 White or Black children between the ages of 9 and 10. Of all participants, 7751 were Whites and 2167 were Blacks. Of all, 815 (9.2%) children had some STB history (Table 2).

### 3.2. Descriptive Data by Race

Table 2 summarizes the descriptive data by race. While age and household size did not differ across groups, parental education and household income were lower in Black than in White children. Compared to White families, Black families were less likely to be married and employed. STBs were also higher in Black than White children.

### 3.3. Pooled Sample Models

In the pooled sample, and in the absence of any interaction, family conflict was associated with higher STBs, an association that remained significant while SES and other confounders were controlled (Model 1). There was an interaction between race and family conflict on STBs, suggesting that the association between family conflict and STBs was stronger in Black than White children (Model 2) (Table 3).

### 3.4. Race-Specific Models

We found that high family conflict was associated with higher STBs for Black and White children. However, *b* was larger for Black than White children (Table 4).

## 4. Discussion

Higher family conflict was associated with a higher count of lifetime STBs. Race and family conflict had interactive rather than additive effects on children’s STBs. The observed association between family conflict and STBs was stronger for Black than White children. In other words, negative family relations may be a more salient factor associated with STBs for Black than White children. That means Black children with STBs should be evaluated for family conflict more aggressively than White children with STBs. Similarly, Black children with family conflict should be evaluated for STBs more aggressively than White children with a similar level of family conflict.

Our first finding on the association between family conflict and STBs overall is in line with past research [67,68,69]. We already know that various types of stress, including family conflict [42], correlate with STBs in children and adults [70]. Some research has shown that family conflict precipitates STBs in children, youth, and adults [71]. Our finding on the link between family conflict and STBs in children can be interpreted with the growing literature on the role of adverse childhood events in shaping children′s outcomes. This literature suggests that all types of childhood trauma are causally linked to undesired outcomes in childhood and adulthood. The effects of ACEs are not limited to suicide or mental health but extend to a wide range of health outcomes such as obesity, physical conditions, and even mortality. For future research, we need to study to what degree ACEs in the family and community contexts reflect or cause family conflict and are the main causal factors in suicidal thoughts and behaviors.

Our second finding is in line with the research suggesting that relationship quality may be more consequential for the mental health of Black than White individuals. For instance, Lincoln and colleagues showed that Blacks are more vulnerable to the effects of positive and negative relationships on mental health, compared to Whites [48]. Similarly, Krause and colleagues have shown that social support in the church setting has more consequences for Blacks than Whites [49,50,51], and another study showed that secular and religious social support are both more strongly associated with depression of Black than White individuals [52]. The same finding is shown by other research as well [5,53]. However, this literature is mostly on adults or even older adults, and few studies have been done on children. The unique contribution of this study is to extend this finding to children’s STBs.

Our second finding contrasts with the minorities’ diminished returns (MDRs) [13,14,15], suggesting that individual- and family-level risk and protective factors have diminished associations with mental health outcomes for Black than White communities. Previous research on MDRs has provided enormous evidence on smaller changes in outcomes as protective and risk factors change, probably due to structural racism and social stratification and other risk factors that are not relevant to Whites [72,73,74,75]. For example, racial discrimination is associated with Black children STBs, which may not be much relevant to White children STBs [76].

Bauermeister has argued that bad is stronger than good [21]. Conflicting relations have a large undesired effect on American children’s STBs, an effect that cannot be explained by SES and confounders [22]. This is important because close relations and family relations almost always accompany supportive and conflicting relationships.

This study established one Black–White difference in STB correlates, being family conflict. Other previous research has shown various White-Black differences in correlates of STBs. For example, parents′ education shows larger protective effects for White than Black children, youth, and adults [77]. This is because socioeconomic status indicators may lose some of their effects due to structural racism [13,78]. These diminished effects of SES on suicide and other high-risk behaviors in Black than White people are attributed to structural racism and segregation. As a result of racism, high SES Black families continue to experience much stress, while high SES White families report very low stress across domains [15]. Similarly, high SES White families have financial security and wealth, while high SES Black families have far less financial security and wealth [79].

Ali and colleagues investigated suicide circumstances among White, Black, and other race/ethnic groups by sex and age. They used the National Violent Death Reporting System (NVDRS) data files from 2006 to 2015 to explore Black–White differences in STBs′ proximal circumstances. All participants were aged 10 years or older. The authors applied hierarchical logistic regression analysis to reveal racial differences in how non-alcohol substance abuse problems, intimate partner problems, and physical health problems correlate with the suicide of race by sex groups. The authors showed that these differences are not due to potential confounders. The authors advocated for the intersectionality of race and sex for suicide interventions and program planning [80].

In one study [81], 5388 White and 759 Black suicidal attempts resulting in hospitalization were compared. Racial groups were compared for sociodemographic factors, medical history, psychiatric disorders, and outcomes (death). The study showed that Blacks and Whites varied by insurance type. While Whites were commonly under private insurance or self-pay, Blacks were commonly under governmental insurance. Blacks with suicide attempts were more likely to be obese, while Whites with suicide attempts were likely to be underweight. The study did not find Black–White differences in age, sex, or psychopathology of suicide attempts [81].

The results may have implications for clinical practice, public health service, and policy related to RTBs in racially diverse children. This paper′s take-home message is that one size does not fit all, and family conflict may be more relevant to White than Black STBs in children. Tailoring interventions and services for groups based on race may have some advantages, mainly because research has shown racial differences in STB correlations [80]. Joe conducted a review and found very few evidence-based STB prevention or treatment strategies for diverse racial groups such as Blacks. They, however, mentioned that crossover effects for multisystemic therapy for reducing the risk for suicide ideation and attempts in Black males might exist. Their review paper suggested that attachment-based family therapy is salient for use as a clinical practice component for Black children and youth treated for STBs [82].

Some recent research has suggested that children′s STBs are higher in Black than White children, which is very concerning [83]. The traditional assumption that males have a higher STBs rate and suicide prevention is less relevant to Black children is not accurate. This means that both Black boys and girls are at STB risk. Growing research suggests that Black children may not be at a lower but a higher risk of STBs than White children [83]. More research is needed on why STBs are more rapidly increasing in Black than White children, how these differences emerge over time, and whether populations differ in mechanisms and methods of suicide exist between Black and White children.

One of the reasons our result is essential is that STBs increase in all children and youth; however, a more rapid increase can be seen in Black children and youth [84]. Our finding provides a new opportunity for understanding the recent trends and the Black and White suicide risk factors. Unfortunately, Black children′s suicide is often overlooked.

Interventions to prevent White and Black children’s STBs should be ongoing, and policymakers and clinicians should be aware that one size does not always fit all. Comprehensive suicide prevention efforts should consider how race and risk factors interact and how risk factors of suicide vary for Black and White children′s STBs. The research on intersectionality and group differences may leverage helpful research-based information to target sub-populations who are at most risk. More research is needed to develop interventions that leverage protective factors. Such research may help us with innovative prevention strategies that minimize the chance of lives lost due to suicide. As race distribution varies in different communities, and as race differences exist in STBs, suicide prevention may be improved if it considers the context of race in the target community.

## 5. Limitations

Our study is not free of limitations. First, due to a cross-sectional design, the study does not allow causal inferences. We controlled for a limited range of confounders. For example, sexual orientation, gender identity, the experience of sexual assault, depression, and parental history of suicide and alcohol and drug use are among predictors of suicidal behaviors in children, which were not examined in this study [85]. The sample was national but not selected at random. So, the results′ generalizability is limited. We also limited this study to White and Black children. Thus, the results are not generalizable to other racial and ethnic minorities such as Latinos, Asian Americans, or Native Americans.

## 6. Conclusions

In 9–10-year-old American children, family conflict and race have interdependent rather than independent effects on STBs, as that the role of family conflict in STBs is stronger in Black than White children. According to our knowledge, this is the first study to show that family conflict has a stronger association with STBs in Black than White children. The results may have implications for preventing or detecting STBs in Black children, which is pressing public health. Still, more research is needed on the topic, particularly on mediators explaining why race variations in STB correlations exist.

## Figures and Tables

**Table 1:** Items used to measure STBs in children.

Measure/Item	Answers	Coding
**Suicidal Thoughts and Behaviors (Measured Using K-SADS-Structured Interview with the Child)**		
Suicidal ideation, Present	No/Yes	0/1
Suicidal ideation, Past	No/Yes	0/1
Suicidal attempt, Present	No/Yes	0/1
Self-injury, intent to die, Present	No/Yes	0/1
Self-injury, thought could die from behavior, Present	No/Yes	0/1
Suicidal ideation thought of method, Present	No/Yes	0/1
Suicidal ideation, intent to act, Present	No/Yes	0/1
Suicidal ideation, specific plan, Present	No/Yes	0/1
Suicidal behavior, made preparations, Present	No/Yes	0/1
Aborted or interrupted suicide attempts, Present	No/Yes	0/1
Method of actual suicide attempt, Present	No/Yes	0/1
Suicide attempt, thought could die, Present	No/Yes	0/1
Self-injury, intent to die, Past	No/Yes	0/1
Self-Injury, thought could die from behavior, Past	No/Yes	0/1
Suicidal ideation thought of method, Past	No/Yes	0/1
Suicidal ideation, intent to act, Past	No/Yes	0/1
Suicidal ideation, specific plan, Past	No/Yes	0/1
Suicidal behavior, made preparations, Past	No/Yes	0/1
Aborted or interrupted suicide attempts, Past	No/Yes	0/1
Number of suicide attempts, Past	No/Yes	0/1
Suicide attempt, method, Past	No/Yes	0/1
Expect could die from suicide attempt, Past	No/Yes	0/1
**Family Conflict (Self-Report; The Family Environment Scale)**		
We fight a lot in our family.	No/Yes	0/1
Family members sometimes get so angry they throw things.	No/Yes	0/1
Family members hardly ever lose their tempers.	No/Yes	0/1
Family members often criticize each other.	No/Yes	0/1
Family members sometimes hit each other.	No/Yes	0/1
Family members rarely become openly angry.	No/Yes	0/1
If there is a disagreement in our family, we try hard to smooth things over and keep the peace.	No/Yes	0/1
Family members often try to one-up or outdo each other.	No/Yes	0/1
In our family, we believe you don′t ever get anywhere by raising your voice.	No/Yes	0/1

**Table 2:** Descriptive statistics overall and by race.

Characteristics	All *n* = 9918		White *n* = 7751		Black *n* = 2167	
	**Mean**	**SD**	**Mean**	**SD**	**Mean**	**SD**
Age (Year)	9.48	0.51	9.48	0.50	9.47	0.51
Parental Educational Attainment *	16.89	2.49	17.25	2.35	15.58	2.55
Household Income *	7.32	2.38	7.86	1.95	5.36	2.70
Family Conflict *	0.28	0.22	0.28	0.22	0.31	0.23
	*n*	%	*n*	%	*n*	%
Ethnicity *						
Non-Latino	8357	84.3	6380	82.3	1977	91.2
Latino	1561	15.7	1371	17.7	190	8.8
Married Household *						
No	3010	30.3	1593	20.6	1417	65.4
Yes	6908	69.7	6158	79.4	750	34.6
Immigrant						
No	9699	97.8	7575	97.7	2124	98.0
Yes	219	2.2	176	2.3	43	2.0
Sex						
Female	4740	47.8	3667	47.3	1073	49.5
Male	5178	52.2	4084	52.7	1094	50.5
Parents Employed *						
No	2835	28.6	2159	27.9	676	31.2
Yes	7083	71.4	5592	72.1	1491	68.8
STBs (Any) *						
No	9103	91.8	7149	92.2	1954	90.2
Yes	815	8.1	602	7.8	213	9.8
STBs (Items Positively Endorsed)						
0.0	9103	91.8	7149	92.2	1954	90.2
1.0	447	4.5	330	4.3	117	5.4
2.0	159	1.6	122	1.6	37	1.7
3.0	74	0.7	52	0.7	22	1.0
4.0	67	0.7	50	0.6	17	0.8
5.0	24	0.2	15	0.2	9	0.4
6.0	13	0.1	11	0.1	2	0.1
7.0	9	0.1	8	0.1	1	0.0
8.0	9	0.1	5	0.1	4	0.2
9.0	7	0.1	4	0.1	3	0.1
10.0	1	0.0	1	0.0	0	0.0
11.0	2	0.0	1	0.0	1	0.0
12.0	2	0.0	2	0.0	0	0.0
15.0	1	0.0	1	0.0	0	0.0

STBs: Suicidal Thoughts and Behaviors, * *p* < 0.05.

**Table 3:** Association between family conflict and STBs in American children by race.

	*b*	SE	95% CI		*p*	*b*	SE	95% CI		*p*
	All Model 1					All M1 + Interactions				
Race (Black)	−0.06	0.07	−0.19	0.07	0.385	−0.25	0.10	−0.45	−0.04	0.018
Ethnicity (Hispanic)	−0.06	0.07	−0.20	0.08	0.386	−0.06	0.07	−0.20	0.08	0.395
Immigrant	−0.48	0.22	−0.90	−0.06	0.026	−0.48	0.22	−0.90	−0.06	0.027
Sex (Male)	0.29	0.05	0.19	0.38	<0.001	0.29	0.05	0.19	0.38	<0.001
Married Household	−0.41	0.06	−0.54	−0.29	<0.001	−0.41	0.06	−0.54	−0.29	<0.001
Parental Employment (Employed)	−0.11	0.06	−0.21	0.00	0.056	−0.11	0.06	−0.21	0.00	0.057
Age (Years)	0.01	0.05	−0.08	0.11	0.781	0.02	0.05	−0.08	0.11	0.732
Parental Education	0.01	0.01	−0.02	0.03	0.623	0.01	0.01	−0.02	0.03	0.624
Family Income	−0.04	0.01	−0.07	−0.01	0.008	−0.04	0.01	−0.07	−0.01	0.012
Family Conflict	0.93	0.10	0.72	1.13	<0.001	0.77	0.12	0.53	1.01	<0.001
Family Conflict × Race (Black)						0.53	0.22	0.09	0.96	0.017

**Table 4:** Association between family conflict and STBs in American children by race.

	*b*	SE	95% CI		*p*	*b*	SE	95% CI		*p*
	Whites					Blacks				
Ethnicity (Hispanic)	−0.16	0.08	−0.31	0.00	0.048	−0.44	0.20	−0.84	−0.04	0.029
Immigrant	−0.59	0.25	−1.09	−0.10	0.019	−0.35	0.41	−1.16	0.46	0.398
Sex (Male)	0.32	0.06	0.20	0.43	<0.001	0.17	0.10	−0.01	0.36	0.068
Married Household	−0.54	0.07	−0.68	−0.41	<0.001	0.04	0.12	−0.19	0.27	0.740
Parental Employment (Employed)	−0.07	0.06	−0.20	0.06	0.293	−0.27	0.11	−0.48	−0.06	0.012
Age (Years)	−0.08	0.06	−0.19	0.03	0.147	0.25	0.09	0.07	0.43	0.006
Parental Education	−0.01	0.01	−0.04	0.02	0.378	0.06	0.02	0.01	0.10	0.015
Family Income	−0.07	0.02	−0.10	−0.03	<0.001	0.01	0.03	−0.04	0.05	0.840
Family Conflict	0.73	0.12	0.49	0.97	<0.001	1.52	0.19	1.15	1.88	<0.001

## Data Availability

Data of the ABCD study are available here https://nda.nih.gov/abcd (accessed on 5 May 2017).

## References

[1] Hoagwood K.E., Cavaleri M.A., Olin S.S., Burns B.J., Slaton E., Gruttadaro D., Hughes R. (2010). Family support in children’s mental health: A review and synthesis. Clin. Child. Fam. Psychol. Rev..

[2] Vahedi A., Krug I., Westrupp E.M. (2019). Crossover of parents’ work-family conflict to family functioning and child mental health. J. Appl. Dev. Psychol..

[3] Hirsch J.K., Barton A.L. (2011). Positive social support, negative social exchanges, and suicidal behavior in college students. J. Am. College Health.

[4] Henry C.S., Stephenson A.L., Hanson M.F., Hargett W. (1993). Adolescent suicide and families: An ecological approach. Adolescence.

[5] Assari S. (2013). Race and Ethnicity, Religion Involvement, Church-based Social Support and Subjective Health in United States: A Case of Moderated Mediation. Int. J. Prev. Med..

[6] Lincoln K.D., Taylor R.J., Chatters L.M., Joe S. (2012). Suicide, negative interaction and emotional support among black Americans. Soc. Psychiatry Psychiatr. Epidemiol..

[7] Lakey B., Cohen S. (2000). Social Support Theory and Measurement.

[8] House J.S., Umberson D., Landis K.R. (1988). Structures and processes of social support. Annu. Rev. Sociol..

[9] Folkman S., Lazarus R.S. (1984). Stress, Appraisal and Coping.

[10] Leavy R.L. (1983). Social support and psychological disorder: A review. J. Commun. Psychol..

[11] Chatters L.M., Taylor R.J., Jayakody R. (1994). Fictive kinship relations in black extended families. J. Comp. Fam. Stud..

[12] Sanders K.W. (1989). Double Jeopardy—The Precarious Status of Women of Color: Issues of Race/Ethnicity and Gender at the Storrs Campus.

[13] Assari S. (2018). Health Disparities due to Diminished Return among Black Americans: Public Policy Solutions. Soc. Issues Policy Rev..

[14] Assari S. (2018). Unequal Gain of Equal Resources across Racial Groups. Int. J. Health Policy Manag..

[15] Assari S. (2020). Understanding America: Unequal Economic Returns of Years of Schooling in Whites and Blacks. World J. Educ. Res..

[16] Ross K.M., Rook K., Winczewski L., Collins N., Dunkel Schetter C. (2019). Close relationships and health: The interactive effect of positive and negative aspects. Soc. Personal. Psychol. Compass.

[17] Akiyama H., Antonucci T., Takahashi K., Langfahl E.S. (2003). Negative interactions in close relationships across the life span. J. Gerontol. Ser. B Psychol. Sci. Soc. Sci..

[18] Swick K.J., Williams R.D. (2006). An analysis of Bronfenbrenner’s bio-ecological perspective for early childhood educators: Implications for working with families experiencing stress. Early Child. Educ. J..

[19] McLoyd V.C. (1998). Socioeconomic disadvantage and child development. Am. Psychol..

[20] Jack G. (2000). Ecological influences on parenting and child development. Br. J. Soc. Work.

[21] Baumeister R.F., Bratslavsky E., Finkenauer C., Vohs K.D. (2001). Bad is stronger than good. Rev. Gen. Psychol..

[22] Mattson R.E., Paldino D., Johnson M.D. (2007). The increased construct validity and clinical utility of assessing relationship quality using separate positive and negative dimensions. Psychol. Assess..

[23] Formoso D., Gonzales N.A., Aiken L.S. (2000). Family conflict and children’s internalizing and externalizing behavior: Protective factors. Am. J. Community Psychol..

[24] Robins L.N., West P.A., Murphy G.E. (1977). The high rate of suicide in older white men: A study testing ten hypotheses. Soc. Psychiatry.

[25] Mellick E., Buckwalter K.C., Stolley J.M. (1992). Suicide among elderly white men: Development of a profile. J. Psychosoc. Nurs. Ment. Health Serv..

[26] Woodbury M.A., Manton K.G., Blazer D. (1988). Trends in US suicide mortality rates 1968 to 1982: Race and sex differences in age, period and cohort components. Int. J. Epidemiol..

[27] Parker L.D., Cantrell C., Demi A.S. (1997). Older adults’ attitudes toward suicide: Are there race and gender differences?. Death Stud..

[28] Rockett I.R., Wang S., Stack S., De Leo D., Frost J.L., Ducatman A.M., Walker R.L., Kapusta N.D. (2010). Race/ethnicity and potential suicide misclassification: Window on a minority suicide paradox?. BMC Psychiatry.

[29] Hoelter J.W. (1979). Religiosity, fear of death and suicide acceptability. Suicide Life Threat Behav..

[30] Stack S., Wasserman I. (2005). Race and method of suicide: Culture and opportunity. Arch. Suicide Res..

[31] Joe S., Clarke J., Ivey A.Z., Kerr D., King C.A. (2007). Impact of Familial Factors and Psychopathology on Suicidality Among African American Adolescents. J. Hum. Behav. Soc. Environ..

[32] Merchant C., Kramer A., Joe S., Venkataraman S., King C.A. (2009). Predictors of multiple suicide attempts among suicidal black adolescents. Suicide Life Threat Behav..

[33] Xie P., Wu K., Zheng Y., Guo Y., Yang Y., He J., Ding Y., Peng H. (2018). Prevalence of childhood trauma and correlations between childhood trauma, suicidal ideation, and social support in patients with depression, bipolar disorder, and schizophrenia in southern China. J. Affect. Disord..

[34] Assari S., Lankarani M.M. (2016). Association Between Stressful Life Events and Depression; Intersection of Race and Gender. J. Racial Ethn Health Disparities.

[35] Assari S., Lankarani M.M. (2016). Stressful Life Events and Risk of Depression 25 Years Later: Race and Gender Differences. Front. Public Health.

[36] Assari S. (2019). Race, Depression, and Financial Distress in a Nationally Representative Sample of American Adults. Brain Sci..

[37] Assari S., Burgard S., Zivin K. (2015). Long-Term Reciprocal Associations Between Depressive Symptoms and Number of Chronic Medical Conditions: Longitudinal Support for Black-White Health Paradox. J. Racial Ethn Health Disparities.

[38] Assari S., Lankarani M.M. (2016). Chronic Medical Conditions and Negative Affect; Racial Variation in Reciprocal Associations Over Time. Front. Psychiatry.

[39] Cobb S., Javanbakht A., Khalifeh Soltani E., Bazargan M., Assari S. (2020). Racial Difference in the Relationship Between Health and Happiness in the United States. Psychol Res. Behav. Manag..

[40] Caucus C.B. (2019). Ring the Alarm: The Crisis of Black Youth Suicide in America.

[41] Sa J., Choe C.S., Cho C.B., Chaput J.P., Lee J., Hwang S. (2020). Sex and Racial/Ethnic Differences in Suicidal Consideration and Suicide Attempts among US College Students, 2011–2015. Am. J. Health Behav..

[42] DeVille D.C., Whalen D., Breslin F.J., Morris A.S., Khalsa S.S., Paulus M.P., Barch D.M. (2020). Prevalence and family-related factors associated with suicidal ideation, suicide attempts, and self-injury in children aged 9 to 10 years. JAMA Netw. Open..

[43] Dumais A., Lesage A.D., Lalovic A., Séguin M., Tousignant M., Chawky N., Turecki G. (2005). Is violent method of suicide a behavioral marker of lifetime aggression?. Am. J. Psychiatry.

[44] Zouk H., Tousignant M., Seguin M., Lesage A., Turecki G. (2006). Characterization of impulsivity in suicide completers: Clinical, behavioral and psychosocial dimensions. J. Affect. Disord..

[45] Rosario-Williams B., Rowe-Harriott S., Ray M., Jeglic E., Miranda R. (2020). Factors precipitating suicide attempts vary across race. J. Am. Coll Health.

[46] Hatch S.L., Dohrenwend B.P. (2007). Distribution of traumatic and other stressful life events by race/ethnicity, gender, SES and age: A review of the research. Am. J. Community Psychol..

[47] Keyes C.L. (2009). The Black-White paradox in health: Flourishing in the face of social inequality and discrimination. J. Pers..

[48] Lincoln K.D., Chatters L.M., Taylor R.J. (2003). Psychological Distress among Black and White Americans: Differential Effects of Social Support, Negative Interaction and Personal Control. J. Health Soc. Behav..

[49] Krause N. (2016). Assessing supportive social exchanges inside and outside religious institutions: Exploring variations among Whites, Hispanics, and Blacks. Soc. Indic. Res..

[50] Krause N., Ironson G. (2017). Positive God images and positive emotions toward God: Exploring variations among Whites, Blacks, and Hispanics. Pastor. Psychol..

[51] Krause N. (2002). Church-based social support and health in old age: Exploring variations by race. J. Gerontol. B Psychol. Sci. Soc. Sci..

[52] Assari S., Moghani Lankarani M. (2018). Secular and Religious Social Support Better Protect Blacks than Whites against Depressive Symptoms. Behav. Sci. Basel.

[53] Reese A.M., Thorpe R.J., Bell C.N., Bowie J.V., LaVeist T.A. (2012). The effect of religious service attendance on race differences in depression: Findings from the EHDIC-SWB study. J. Urban. Health.

[54] Gordon J.L., Johnson J., Nau S., Mechlin B., Girdler S.S. (2017). The role of chronic psychosocial stress in explaining racial differences in stress reactivity and pain sensitivity. Psychosomatic Med..

[55] Assari S. (2020). Parental Education and Spanking of American Children: Blacks’ Diminished Returns. World J. Educ. Res..

[56] Magnus K.B., Cowen E.L., Wyman P.A., Fagen D.B., Work W.C. (1999). Correlates of resilient outcomes among highly stressed African—American and White urban children. J. Community Psychol..

[57] Joe S., Niedermeier D.M. (2008). Social work research on African Americans and suicidal behavior: A systematic 25-year review. Health Soc. Work.

[58] Langhinrichsen-Rohling J., Friend J., Powell A. (2009). Adolescent suicide, gender, and culture: A rate and risk factor analysis. Aggress. Violent Behav..

[59] (2018). Alcohol Research: Current Reviews Editorial, S. NIH’s Adolescent Brain Cognitive Development (ABCD) Study. Alcohol. Res..

[60] Casey B.J., Cannonier T., Conley M.I., Cohen A.O., Barch D.M., Heitzeg M.M., Soules M.E., Teslovich T., Dellarco D.V., Garavan H. (2018). The Adolescent Brain Cognitive Development (ABCD) study: Imaging acquisition across 21 sites. Dev. Cogn. Neurosci..

[61] Garavan H., Bartsch H., Conway K., Decastro A., Goldstein R.Z., Heeringa S., Jernigan T., Potter A., Thompson W., Zahs D. (2018). Recruiting the ABCD sample: Design considerations and procedures. Dev. Cogn. Neurosci..

[62] Auerbach R.P., Chase H.W., Brent D.A. (2021). The Elusive Phenotype of Preadolescent Suicidal Thoughts and Behaviors: Can Neuroimaging Deliver on Its Promise?.

[63] Vidal-Ribas P., Janiri D., Doucet G.E., Pornpattananangkul N., Nielson D.M., Frangou S., Stringaris A. (2021). Multimodal neuroimaging of suicidal thoughts and behaviors in a US population-based sample of school-age children. Am. J. Psychiatry.

[64] Boyd C.P., Gullone E., Needleman G.L., Burt T. (1997). The Family Environment Scale: Reliability and normative data for an adolescent sample. Fam. Process..

[65] Moos R.H. (1994). Family Environment Scale Manual: Development, Applications, Research.

[66] Auchter A.M., Hernandez Mejia M., Heyser C.J., Shilling P.D., Jernigan T.L., Brown S.A., Tapert S.F., Dowling G.J. (2018). A description of the ABCD organizational structure and communication framework. Dev. Cogn Neurosci..

[67] Shagle S.C., Barber B.K. (1993). Effects of family, marital, and parent-child conflict on adolescent self-derogation and suicidal ideation. J. Marriage Fam..

[68] Campbell N.B., Milling L., Laughlin A., Bush E. (1993). The psychosocial climate of families with suicidal pre-adolescent children. Am. J. Orthopsychiatry.

[69] Asarnow J.R., Carlson G.A., Guthrie D. (1987). Coping strategies, self-perceptions, hopelessness, and perceived family environments in depressed and suicidal children. J. Consult. Clin. Psychol..

[70] Cerel J., Fristad M.A., Weller E.B., Weller R.A. (2000). Suicide-bereaved children and adolescents: II. Parental and family functioning. J. Am. Acad. Child. Adolesc. Psychiatry.

[71] Brent D.A., Mann J.J. (2006). Familial pathways to suicidal behavior-understanding and preventing suicide among adolescents. N. Engl. J. Med..

[72] Assari S., Bazargan M., Caldwell C. (2019). Parental Educational Attainment and Chronic Medical Conditions among American Youth; Minorities’ Diminished Returns. Children.

[73] Assari S., Boyce S., Bazargan M., Caldwell C.H., Zimmerman M.A. (2020). Place-Based Diminished Returns of Parental Educational Attainment on School Performance of Non-Hispanic White Youth. Front. Educ..

[74] Assari S., Caldwell C., Bazargan M. (2020). Parental educational attainment and relatives’ substance use of American youth: Hispanics Diminished Returns. J. Biosci. Med. Irvine.

[75] Boyce S., Bazargan M., Caldwell C.H., Zimmerman M.A., Assari S. (2020). Parental Educational Attainment and Social Environmental of Urban Public Schools in the U.S.: Blacks’ Diminished Returns. Children.

[76] Assari S., Moghani Lankarani M., Caldwell C.H. (2017). Discrimination Increases Suicidal Ideation in Black Adolescents Regardless of Ethnicity and Gender. Behav. Sci..

[77] Assari S., Schatten H.T., Arias S.A., Miller I.W., Camargo C.A., Boudreaux E.D. (2019). Higher Educational Attainment is Associated with Lower Risk of a Future Suicide Attempt Among Non-Hispanic Whites but not Non-Hispanic Blacks. J. Racial Ethn Health Disparities.

[78] Assari S., Caldwell C.H., Mincy R.B. (2018). Maternal Educational Attainment at Birth Promotes Future Self-Rated Health of White but Not Black Youth: A 15-Year Cohort of a National Sample. J. Clin. Med..

[79] Assari S. (2020). College Graduation and Wealth Accumulation: Blacks’ Diminished Returns. World J. Educ. Res..

[80] Ali B., Rockett I., Miller T. (2019). Variable Circumstances of Suicide Among Racial/Ethnic Groups by Sex and Age: A National Violent-Death Reporting System Analysis. Arch. Suicide Res..

[81] Assari S. (2018). Suicide Attempts in Michigan HealthCare System; Racial Differences. Brain Sci..

[82] Joe S., Scott M.L., Banks A. (2018). What Works for Adolescent Black Males at Risk of Suicide: A Review. Res. Soc. Work Pract..

[83] Martínez-Alés G., Pamplin J.R., Rutherford C., Gimbrone C., Kandula S., Olfson M., Gould M.S., Shaman J., Keyes K.M. (2021). Age, period, and cohort effects on suicide death in the United States from 1999 to 2018: Moderation by sex, race, and firearm involvement. Mol. Psychiatry.

[84] Shain B.N. (2019). Increases in rates of suicide and suicide attempts among black adolescents. Pediatrics.

[85] Baiden P., LaBrenz C.A., Asiedua-Baiden G., Muehlenkamp J.J. (2020). Examining the intersection of race/ethnicity and sexual orientation on suicidal ideation and suicide attempt among adolescents: Findings from the 2017 Youth Risk Behavior Survey. J. Psychiatr Res..

